# Development of a Multi-Step Leukemogenesis Model of MLL-Rearranged Leukemia Using Humanized Mice

**DOI:** 10.1371/journal.pone.0037892

**Published:** 2012-06-20

**Authors:** Kunihiko Moriya, Makiko Suzuki, Yohei Watanabe, Takeshi Takahashi, Yoko Aoki, Toru Uchiyama, Satoru Kumaki, Yoji Sasahara, Masayoshi Minegishi, Shigeo Kure, Shigeru Tsuchiya, Kazuo Sugamura, Naoto Ishii

**Affiliations:** 1 Department of Microbiology and Immunology, Tohoku University Graduate School of Medicine, Sendai, Japan; 2 Department of Pediatrics, Tohoku University Graduate School of Medicine, Sendai, Japan; 3 Department of Medical Genetics, Tohoku University Graduate School of Medicine, Sendai, Japan; 4 Central Institute for Experimental Animals, Kawasaki, Japan; 5 Division of Blood Transfusion, Tohoku University Hospital, Sendai, Japan; 6 Miyagi Cancer Center, Natori, Japan; Emory University, United States of America

## Abstract

Mixed-lineage-leukemia (MLL) fusion oncogenes are intimately involved in acute leukemia and secondary therapy-related acute leukemia. To understand MLL-rearranged leukemia, several murine models for this disease have been established. However, the mouse leukemia derived from mouse hematopoietic stem cells (HSCs) may not be fully comparable with human leukemia. Here we developed a humanized mouse model for human leukemia by transplanting human cord blood-derived HSCs transduced with an MLL-AF10 oncogene into a supra-immunodeficient mouse strain, NOD/Shi-*scid*, IL-2Rγ^−/−^ (NOG) mice. Injection of the MLL-AF10-transduced HSCs into the liver of NOG mice enhanced multilineage hematopoiesis, but did not induce leukemia. Because active mutations in ras genes are often found in MLL-related leukemia, we next transduced the gene for a constitutively active form of K-ras along with the MLL-AF10 oncogene. Eight weeks after transplantation, all the recipient mice had developed acute monoblastic leukemia (the M5 phenotype in French-American-British classification). We thus successfully established a human MLL-rearranged leukemia that was derived *in vivo* from human HSCs. In addition, since the enforced expression of the mutant K-ras alone was insufficient to induce leukemia, the present model may also be a useful experimental platform for the multi-step leukemogenesis model of human leukemia.

## Introduction

Chromosomal translocations that result in MLL (mixed-lineage leukemia)-fusion oncogenes are associated with acute myeloid leukemia (AML), acute lymphoblastic leukemia (ALL), myelodysplastic syndrome, and secondary therapy-related acute leukemia [1]. More than 60 partner genes that form MLL-fusion oncogenes have been identified [2], and the partner usually characterizes the specific pathological phenotype of the MLL-rearranged leukemia. For example, MLL-rearranged AMLs, more than 60% of which involve the MLL-AF9, MLL-AF10, or MLL-AF6 fusion gene, often exhibit the morphological subtypes acute myelomonoblastic leukemia and monoblastic leukemia, which are classified as French-American-British (FAB) M4 and M5, respectively [3]. Although patients with MLL-rearranged leukemia usually show a poor prognosis and are sometimes resistant to therapy [3], [4], the development of efficient therapies targeting the MLL-fusion genes has been slow, in part because much is still unknown about the cellular and molecular mechanisms underlying this disease.

To gain insight into the etiology and pathogenesis of MLL-rearranged leukemia, several mouse models have been developed. Mice with a knocked-in MLL-AF9 fusion gene develop AML as they age [5], although in humans the congenital MLL-AF9 rearrange typically affects infants. Leukemia has also been generated in mice by exogenously expressing an MLL-fusion gene in the animals or by transplanting mouse hematopoietic stem cells (HSCs) that have been retrovirally transduced with an MLL-fusion gene [6]. However, the phenotype and pathogenesis of the leukemia produced in these murine models sometimes do not match those observed in human leukemia associated with the same genetic lesion. For example, the MLL-ENL fusion gene is frequently associated with B-precursor ALL in humans, but generates AML in mice [7]. Thus, there is a gap in our understanding about mouse versus human leukemogenesis, and further leukemia modeling studies using human primary leukemia cells are needed [8].

*In vivo* studies in which primary human leukemia cells were transplanted into immunodeficient mice, provided significant advances in our understanding of the pathogenesis of human leukemia [9], [10]. However, these models, in which the leukemia cells had already developed in human patients before being transplanted into mice, are not suitable for studying physiological leukemogenesis, including the initiation and progression processes since these processes do not occur in the model. Therefore, a new experimental model for analyzing leukemogenesis in which primary human HSCs are converted into leukemia cells by genetic hits is needed.

In the past two decades, a number of studies have tried to graft human HSCs into immunodeficient mice, but these attempts to reconstitute human blood cells in mice have failed [11]. However, the recent development of a new immunodeficient mouse strain has opened up the possibility for generating better xenotransplantation systems. NOD/SCID mice with a targeted mutation of the IL-2-receptor γ chain gene (NOD/Shi-*sci*d IL-2Rγ^−/−^ (NOG) and NOD/LtSz-scid IL-2Rγ^−/−^ (NSG) mice) lack T, B, and NK cells and some innate immunity functions [11], [12]. When human HSCs are transplanted into these mice, human T, B, NK, and dendritic cells develop normally and are maintained in the mice for at least 10 months[13]–[17]. Thus, these immunodeficient mice may be good recipients for examining, not only the normal development of human HSCs, but also their abnormal development, including leukemogenesis. Two previous studies modeled the initiation and progression of human acute leukemia by ectopically expressing MLL-fusion genes in human HSCs, and transplanting these cells into special immunodeficient mice [18], [19]. Dick et al demonstrated that ectopic expression of AF9 or MLL-ENL in human HSCs caused leukemia in NOD/SCID mice in which the NK cells were depleted by an anti-NK cell antibody [18]. Similarly, Mulloy et al successfully modeled acute leukemia by transducing MLL-AF9 into human HSCs, and transplanting these cells into NOD/SCID mice that expressed transgenic human SCF, GM-CSF, and IL-3 genes [19]. However, in a similar model using NSG mice, the enforced expression of MLL-AF4 in HSCs did not induce leukemia [20].

Despite intensive studies on MLL-ENL, MLL-AF4, and MLL-AF9, there has been no report investigating the *in vivo* oncogenic potential of MLL-AF10 in human HSCs, even though MLL-AF10 is the second most common MLL rearrangement (13%) in pediatric MLL-rearranged AML [3]. Here, using NOG mice, we demonstrated that MLL-AF10 could enhance the multilineage hematopoiesis of human HSCs, even though the MLL-AF10 in mouse HSCs preferentially enhances myeloid differentiation [21], [22]. More importantly, the co-transfection of MLL-AF10 with an active form of the K-ras gene induced acute monoblastic leukemia with the FAB M5 phenotype. These results provide a novel leukemia model using human cells showing the requirement of two genetic hits for leukemogenesis.

## Methods

All procedures were performed according to the protocols approved by the Institutional Committee for Use and Care of Laboratory Animals of Tohoku University, which was granted by Tohoku University Ethics Review Board (No. 2010MA165) and the Guide for Care and Use of Laboratory Animals published by the U.S. National Institutes of Health (NIH publication 85-23, revised 1996).

### CD34^+^ Hematopoietic Stem Cell Isolation

Cord blood from full-term human deliveries was obtained from the Miyagi Cord Blood Bank (Miyagi, Japan) and RIKEN Bioresource Center Cell Bank (Tsukuba, Japan), following the institutional guidelines approved by the Tohoku University Committee on Clinical Investigations. Mononuclear cells were isolated from the cord blood by density gradient centrifugation using Lymphocyte Separation Medium (MP Biomedicals, Solon, OH, USA) after removing the phagocytes with silica (Immuno Biological Laboratories, Takasaki, Japan). The cells were washed and suspended in PBS containing 2%FCS. CD34^+^ stem cells were obtained by magnetic cell sorting (MACS) (Miltenyi Biotech, Bergisch Gladbach, Germany). Briefly, CD34^+^ cells were labeled with a biotin-conjugated anti-human CD34 monoclonal antibody (Serotec, Oxford, UK) after blocking the Fc receptor. The cells were then incubated with anti-biotin Microbeads (Miltenyi Biotech). The magnetically labeled CD34^+^ cells were purified twice on LS columns (Miltenyi Biotech). The purity of the CD34^+^ fraction was >95%. The purified CD34^+^ cells were suspended in Cell Banker (Juji Field, Tokyo, Japan) and cryopreserved at −80°C in a deep freezer until use.

### Plasmid Construction

The retroviral vector, pDΛNsam-IRES-EGFP, which is based on Murine Stem Cell Virus with enhanced green fluorescent protein (EGFP) as a marker under an internal ribosomal entry site (IRES), was kindly provided by Dr. Masafumi Onodera (Tsukuba University) [23]. PLAT-F, a package cell line that produces a pseudotype virus with an RD114 envelope, was previously established and provided by Dr. Toshio Kitamura (Institute of Medical Science, University of Tokyo) [24]. The human MLL-AF10 cDNA [22], which was kindly provided by Dr. M.L. Cleary (Stanford University), was inserted into a cloning site in the pDΛNsam-IRES-EGFP vector to make the pDΛNsam-MLL-AF10 plasmid. To construct the pDΛNsamIRES VENUS vector, the EGFP cDNA of the retroviral vector was replaced with Venus cDNA [25], which was provided by Dr. Hiroyuki Miyoshi (RIKEN, Tsukuba). The human flag-tagged K-ras^G12V^ cDNA, which was previously described [26], was inserted into the Xho I site in the cloning site of pDΛNsamIRES Venus to make pDΛNsam-K-ras^G12V^.

### Gene Delivery into CD34^+^ Cells by Retrovirus

The CD34^+^ cells isolated from cord blood were cultured in X-VIVO15 (Cambrex Bioscience, Walkersville, MD, USA), supplemented with 1% human serum albumin (HSA) (Kaketsuken, Kumamoto, Japan) and stimulated with a cytokine cocktail [100 ng/ml stem cell factor, 100 ng/ml Flt-3 ligand, 100 ng/ml thrombopoietin, and 100 ng/ml IL-6 (Peprotech)] in a 24-well plate (2×10^5^ per well) for 48 h. The stimulated CD34^+^ cells were then harvested and placed into non-tissue culture-treated 6-well plates (Becton Dickinson) that had been coated with 20 µg/ml CH-296, a recombinant fibronectin fragment (Retronectin, Takara, Japan). (3×10^5^ cells per well) in the presence of the respective virus supernatant. The virus supernatants were diluted 1∶2 with X-VIVO15 containing 1% HSA and the cytokine cocktail described above. Every 12 h, the medium was replaced with fresh virus supernatant. After 48 h of culture, the frequency of GFP- and/or Venus-expressing CD34^+^ cells was examined by FACS.

**Figure 1:**
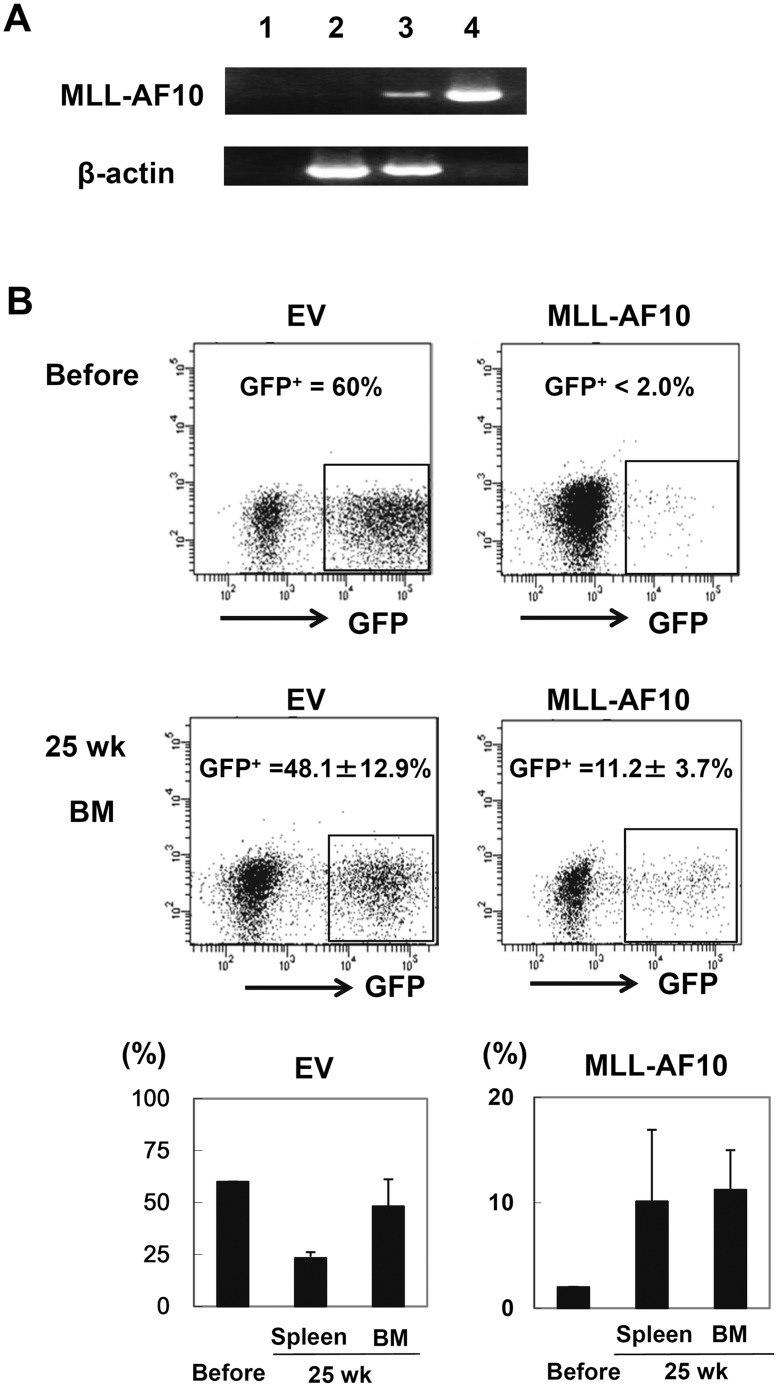
Enforced expression of MLL-AF10 augmented multilineage hematopoiesis, but was insufficient to induce leukemogenesis in vivo. (A) Representative RT-PCR results confirming the long-term expression of the MLL-AF10 transcript in the BM cells of mice 25 weeks after transplantation (lane 1; water, lane 2; cells from a mouse in the EV-transfused group, lane 3; cells from a mouse in the MLL-AF10-transfused group, and lane 4; positive control (MLL-AF10 plasmid)). (B) Flowcytometric analysis of the frequency of GFP^+^ cells. The indicated vector (EV, left or MLL-AF10, right)-transduced human CD34^+^ cells, whose *in vitro* GFP expression is shown in the upper panels (Before) of the flowcytometric analysis, were transplanted into NOG mice. Twenty-five weeks later, the GFP-expressing cells gated on human CD45^+^ hematopoietic cells in the BM was measured (lower panels of the FACS profiles). The data shown are representative of 3 independent experiments. The graphs show the frequency of GFP^+^ cells in human CD34^+^ cells just before transplantation (Before) and the mean ± SD of the frequency of GFP^+^ cells in the BM and spleen of mice receiving transplants of EV-transduced HSCs (n = 8) or of MLL-AF10-transduced HSCs (n = 6) 25 weeks after transplantation, in one representative experiment of three. Similar results were obtained in the 3 independent experiments.

### Cell Transplantation into Mice

NOD/Shi-*scid*, IL-2Rγ^−/−^ (NOG) mice were obtained from the Central Institute for Experimental Animals (CIEA, Kawasaki, Japan) and maintained in an animal facility at Tohoku University Graduate School of Medicine under specific pathogen free conditions. All procedures were performed according to the protocols approved by Tohoku University Ethics Review Board (No. 2010MA165). Neonatal (1–3 days) NOG mice were irradiated with 30 cGy of X-rays, and the cultured CD34^+^ cells (1.0−1.5×10^5^) in 70 µl PBS were intrahepatically injected into the mice later the same day. From one cord blood sample, donor cells could be prepared for transplantation into 3 recipient mice. Therefore, 2 to 4 different cord blood samples were used for each mouse group. Since even NOG mouse sometimes failed to engraft human HSCs, 9 recipient mice that had no human CD45^+^ hematopoietic cell in the BM and spleen at the sacrifice were excluded from this study.

### Antibodies and Flow Cytometric Analysis

Surface markers were detected with the following fluorescent human specific antibodies from BD Biosciences, San Jose, CA, USA: CD33-PE, HLA-DR-PE, CD14-PE, CD19-APC, CD11b-APC, CD15-APC, and CD45-APC-Cy7. To monitor the reconstitution of human blood in NOG mice, peripheral blood was periodically taken from the tail vein. To examine the spleen, bone marrow (BM), and liver, single-cell suspensions were prepared from the spleen and liver by mincing with metal mesh and from the BM by flushing the tibiae and femurs with PBS containing 2% FCS, using a 27-gauge needle. The cells were stained with the relevant antibodies for 15 min on ice, then washed with cold PBS containing 2% FCS, and stained with the appropriate secondary antibodies when necessary. After the final wash, the cells were subjected to flowcytometric analysis with a FACSCanto II cytometer (BD Biosciences). To distinguish between the EGFP (510 nm) and Venus (535 nm) fluorescences, an apporopiate combination of mirrors and optical filters was used. The proportion of each lineage was calculated using FACS Diva software (BD Biosciences).

### Reverse Transcription-PCR (RT-PCR)

Total RNA was prepared from cells using Trizol Reagent (Invitrogen, Carlsbad, CA, USA). The concentration of total RNA was measured by a NanoDrop 1000 (Nanodrop Technologies Inc., Wilmington, DE, USA), and first-strand cDNA was synthesized using Superscript II (Invitrogen) with an oligo (dT) primer. The cDNA for MLL-AF10, Flag-K-ras, or β-actin was amplified with specific primers using Ex-Taq polymerase (Takara). The primer sets were as follows; MLL-AF10: forward, 5̀- ccaaaagaaaaggaaaaacca and reverse, 5̀-gacttcagcattcctaaaatgtca; Flag-K-ras: forward, 5̀-gactacaaagacgatgacg and reverse, 5̀-ccctgtcttgtctttgc; β-actin: forward, 5̀-gctcgtcgtcgacaacggctc and reverse, 5̀-caaacatgatctgggtcatcttctc.

### DNA Extraction and Southern Blot Analysis

Genomic DNA was extracted from spleens using the GenElute™ Mammalian Genomic DNA Miniprep kit (Sigma-Aldrich, USA). For Southern blot analyses, 10 µg genomic DNA was digested with BglII (Toyobo, Osaka, Japan) overnight at 37°C and separated by electrophoresis on a 0.7% agarose gel for 12 h at 40 V. The DNA was transferred overnight to a Hybond N^+^ membrane (GE Healthcare, Chalfont St. Giles, UK). The DNA was fixed onto the membrane with a UV Stratalinker 1800 (Stratagene). The membrane was probed with 10^6^ cpm/ml of [C^32^P]dCTP (Perkin Elmer)-labeled EGFP cDNA. This EGFP probe was derived from a 700-bp ScaI fragment of the pDΛNsam-IRES-EGFP vector, and radiolabeled using a random prime labeling kit (Takara). The membrane was then washed and exposed to a BAS-III imaging plate (Fuji Photo Film Co., LTD, Japan), and the image was produced and analyzed using a BAS-III software (Fuji Photo Film Co., LTD).

### Histopathology

The BM, spleen, and liver were examined macroscopically, and the spleen was weighed. Tissues were fixed in 10% formalin for at least 48 hours. Hematoxylin and eosin staining or May-Giemsa staining was performed on 3-µm sections using a routine protocol. For CD45 immunostaining, after dewaxing and antigen retrieval by microwave, the sections were incubated for 1 h with mouse anti-human CD45 (Dako Japan, Tokyo). Immunodetection was performed using HRP as the visualization enzyme and DAB as the substrate chromogen. All slides were examined by light microscopy using an Olympus IX50 microscope (Olympus, Tokyo, Japan) and a SPOT camera.

### Statistical Analysis

Data are presented as the mean ± standard deviation (SD). Statistical analyses were performed using either a paired or unpaired Student’s t-test. Statistical significance was defined as a P-value <0.01 (**).

## Results

### Enforced Expression of MLL-AF10 Augmented Multilineage Hematopoiesis, but was Insufficient for Leukemogenesis in vivo

To examine the pathogenesis of the MLL-AF10 fusion gene during the differentiation of human blood cells from HSCs, MLL-AF10-infected cord blood CD34^+^ cells were transplanted intrahepatically into sublethally irradiated neonatal NOG mice. None of the recipient mice showed any sign of disease, such as body weight loss or the appearance of abnormal peripheral leukocytes, 25 weeks after transplantation (data not shown). The expression of the transduced MLL-AF10 gene in the reconstituted human blood cells was confirmed (Figure 1A), indicating that the enforced expression of MLL-AF10 in human HSCs could not induce any hematological disorders including leukemia in this model. However, the MLL-AF10 appeared to augment hematopoiesis. As shown in Figure 1B, when empty control vector (EV) was transdused into human CD34^+^ HSCs, the frequency of the GFP^+^ population did not change significantly (60% before transplantation and 24% and 48% after transplantation in the spleen and BM, respectively) (Figure 1B). In contrast, when MLL-AF10 was exogenously expressed, the GFP^+^ population markedly increased from 2% (before) to 11% (25 weeks after transplantation) (Figure 1B). Therefore, the MLL-AF10-positive blood cells may have a growth advantage *in vivo* compared to the MLL-AF10-negative normal blood cells (CD45^+^GFP^-^ cells).

**Figure 2:**
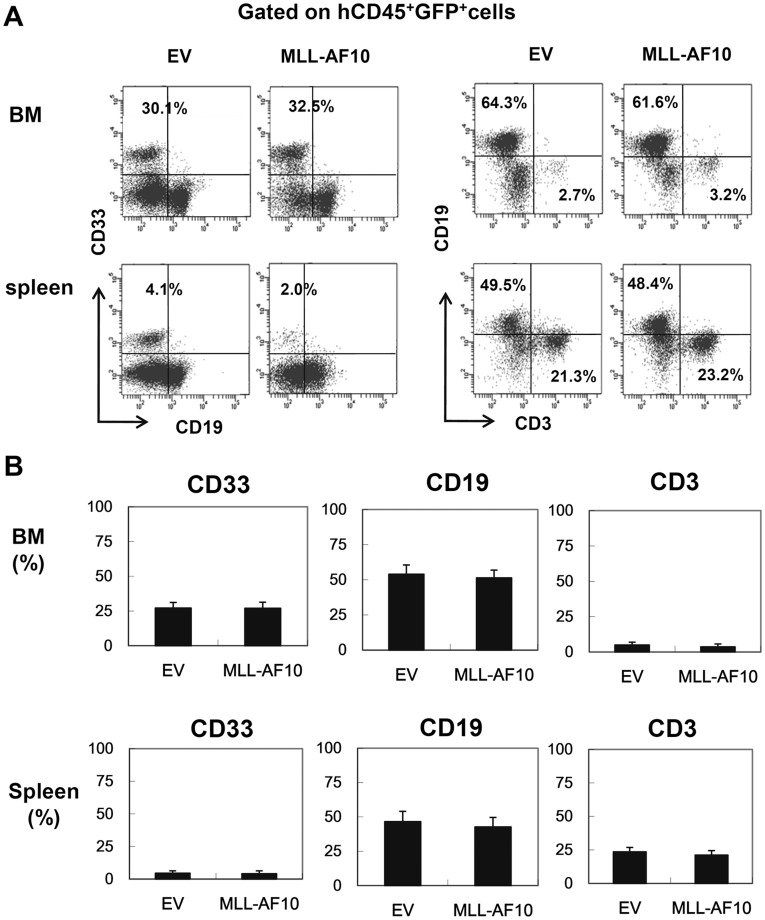
Flowcytometric analysis confirming multilineage engraftment. (A) Representative flowcytometric results of EV- or MLL-AF10-transduced human hematopoietic cells. The human CD45^+^ GFP^+^ cells were analyzed for their lineage distributions to B cells (CD19^+^), T cells (CD3^+^), and myeloid cells (CD33^+^). (B) Multilineage differentiation of MLL-AF10-transduced cells. The data shows cells gated on the CD45^+^GFP^+^ cell population. The graph represents the mean ± SD of the frequencies of CD33^+^ myeloid cells, CD19^+^ B cells, and CD3^+^ T cells in the BM (upper) and spleens (lower) of mice engrafted with EV-transduced (n = 8) or MLL-AF10-transduced (n = 6) CD34^+^ HSCs. No difference in the graft composition between the EV- and MLL-AF10-expressing CD34^+^ HSCs was found. Similar results were obtained in 3 independent experiments.

We next characterized the lineage composition of the human CD45^+^GFP^+^ cells in the BM and spleen of both groups. The GFP^+^ MLL-AF10-expressing cells did not show a skewed lineage differentiation compared to the EV-transfected hematopoietic cells (Figures 2A and 2B). In contrast to the previous reports with mouse HSCs, which showed skewed myeloid differentiation by the exogenous expression of MLL-AF10 [21], [22], the enforced expression of MLL-AF10 in human HSCs enhanced the hematopoietic repopulation of HSCs without affecting cell differentiation.

**Figure 3:**
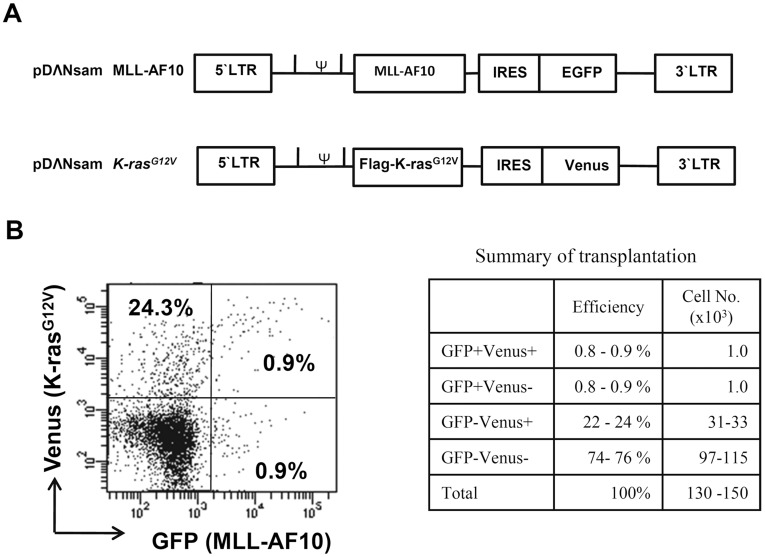
Co-transduction of activated K-ras and MLL-AF10 into CD34^+^HSCs. (A) Schematic structure of the MLL-AF10-GFP and Flag-K-ras^G12V^-Venus vectors. (B) Infectious efficiency of the MLL-AF10-GFP and Flag-K-ras^G12V^-Venus co-transfection. The data and the summary shown in the flowcytometric analysis is representative of the transduced CD34^+^ HSCs in 2 experiments.

**Figure 4:**
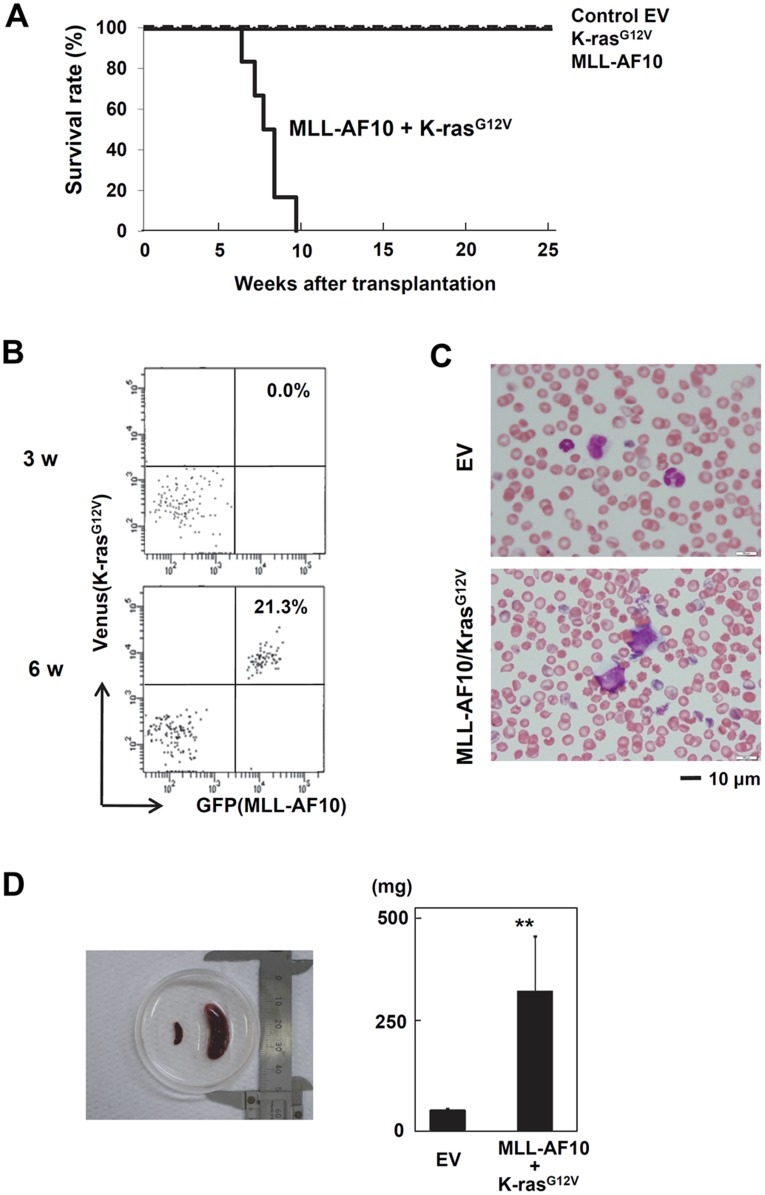
Cooperation of MLL-AF10 with activated K-ras induced acute monoblastic leukemia. (A) Kaplan-Meier survival analysis of mice receiving transplants of human HSCs transfected with EV (n = 8), K-ras^ G12V^ (n = 12), MLL-AF10 (n = 6), or MLL-AF10 plus K-ras^G12V^ (n = 6) vectors. (B) GFP and Venus expression in peripheral blood cells at the indicated weeks after transplantation with human HSCs co-transfected with the MLL-AF10 and K-ras^G12V^ genes. (C) May-Giemsa staining of the peripheral blood of mice engrafted with human HSCs co-transfected with the MLL-AF10 and K-ras^G12V^ genes. Morphologic leukemia cells were found in the peripheral blood of these mice 50 days after transplantation. (D) Splenomegaly in the MLL-AF10/K-ras^G12V^ mice. Spleens from mice engrafted with EV-transduced HSCs (left) and MLL-AF10/K-ras^G12V^ co-transduced HSCs (right) are shown. The graph shows the mean ± SD of the spleen weights from mice receiving transplants of EV-transduced HSCs (n = 6) or of MLL-AF10/K-ras^G12V^ co-transduced HSCs (n = 6). ** represents p<0.01.

**Figure 5:**
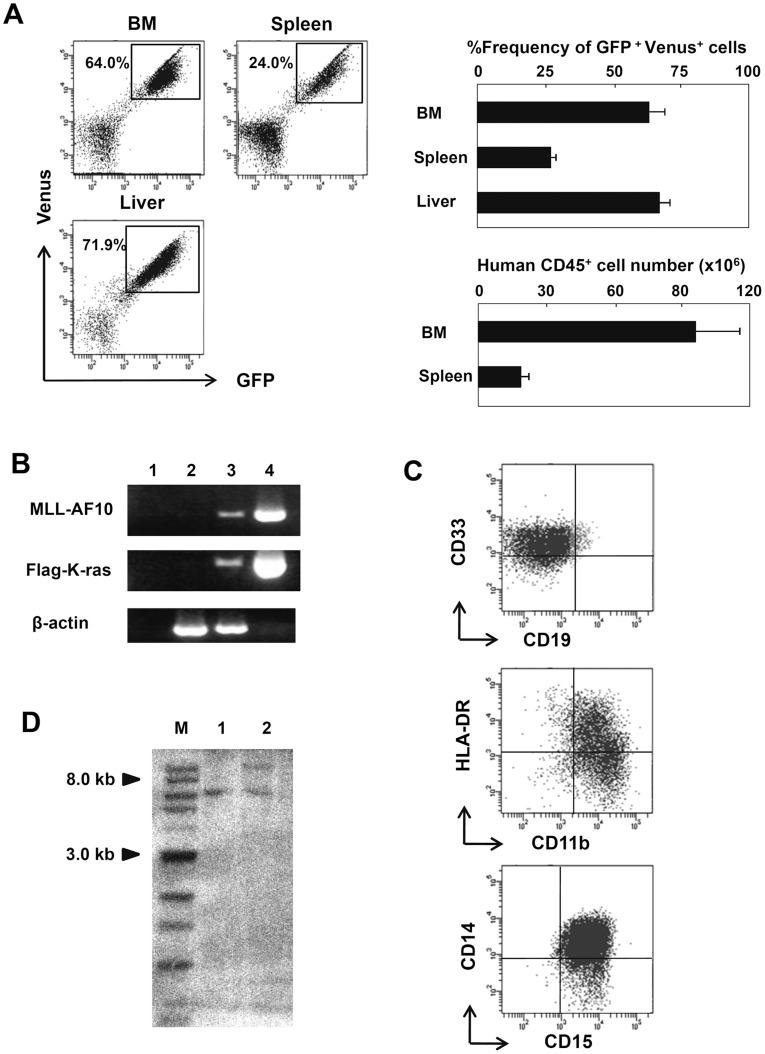
Immunophenotype and clonality of the MLL-AF10/K-ras-induced leukemia. (A) Frequencies of GFP^+^/Venus^+^ cells or human CD45^+^ cells in the BM, spleen, and liver at 8 weeks after transplantation with human HSCs co-transfected with the MLL-AF10 and K-ras^G12V^ genes were examined by flowcytometric analysis. The flowcytometry data shown are representative of 6 to 8 mice per group in one representative experiment of two (left). The average of %frequencies of the GFP^+^ and Venus^+^ cells in whole cells in the indicated organs is shown with the standard deviation (right, upper; n = 6). The absolute cell number of human CD45^+^ cells in the indicated organs is shown with the standard deviation (right, lower; n = 6). (B) Representative RT-PCR results confirming the stable, long-term expression of the MLL-AF10 and Flag-K-ras^G12V^ transcripts in human hematopoietic cells in the BM of mice 8 weeks after transplantation. (C) Lineage distribution of the GFP^+^ and Venus^+^ cells in the BM of a mouse engrafted with HSCs expressing MLL-AF10 and activated K-ras. (D) Southern blot analysis of DNA prepared from the human blood cells in the spleen of mice receiving transplants of MLL-AF10/K-ras^G12V^ co-transduced HSCs. Independent leukemia samples derived from two mice (lane 1; mouse 1 and lane 2; mouse 2) were examined. DNA was digested with Bgl II and probed with an EGFP probe. M: marker.

### Co-transduction of Activated K-ras and MLL-AF10 into CD34^+^ HSCs

As described above, transduction of the MLL-AF10 gene alone was not sufficient to induce leukemogenesis from human HSCs in the present model. Similarly, Menendez et al recently demonstrated that the MLL-AF4 gene alone cannot induce leukemia from human CD34^+^ HSCs in a humanized mouse model [20]. These observations prompted us to generate a two-hit model of leukemogenesis using MLL-AF10 and one additional oncogene. We thus used the K-ras^G12V^ oncogene because K-ras^G12V^ is a very well-characterized oncogene, and because ras mutations are found in about 30% of the cases of pediatric MLL-rearranged AML [3]. To evaluate two hits in one cell, we needed to distinguish the MLL-AF10 and K-ras^G12V^ expressions. We thus retrovirally transfected CD34^+^ HSCs with MLL-AF10-EGFP and K-ras^G12V^ that was co-expressed with the Venus fluorescent protein, which can be distinguished from EGFP by flowcytometry (Figure 3A). The MLL-AF10 and K-ras co-infected (GFP^+^Venus^+^) cells were quite rare (0.9%) (Figure 3B). Nevertheless, we injected the CD34^+^ HSCs, which contained 74% non-transduced, 0.9% MLL-AF10-alone-transduced, 24% K-ras^G12V^-alone-transduced, and 0.9% co-transduced cells, intrahepatically into NOG mice (Figure 3B).

**Figure 6:**
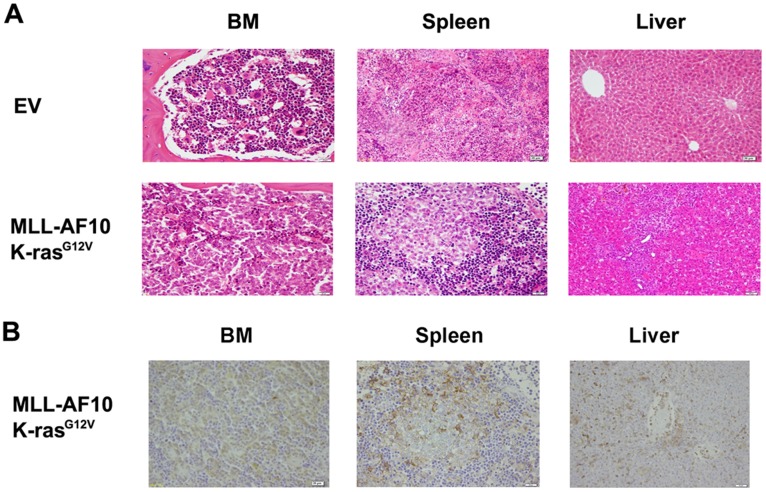
Pathological phenotypes of the leukemia. (A) Hematoxylin and eosin staining showing the infiltration of leukemic cells in the indicated organs of mice engrafted with HSCs expressing the MLL-AF10 and K-ras^G12V^ genes compared to control mice. (B) Immunostaining by a human CD45 mAb in the BM, spleen, and liver in mice engrafted with HSCs expressing the MLL-AF10 and K-ras^G12V^ genes.

### Cooperation of MLL-AF10 with Activated K-ras Induced Acute Monoblastic Leukemia

By 8 weeks after transplantation, several mice in the MLL-AF10/K-ras^G12V^ co-transduced group showed a rough coat, slow movement, and weight loss, while no mice in the other groups (EV, MLL-AF10 alone, and K-ras^G12V^ alone) demonstrated any disease manifestations (data not shown). As the mice in the co-transduced group got sick, most of their peripheral leukocytes became double positive for GFP and Venus (Figure 4B). In addition, morphologically identifiable leukemia cells, characterized by abnormal nuclei and cytoplasmic vacuoles, were found in the peripheral blood (Figure 4C). The morphology of the leukemia cells was compatible with that of the M5 type of FAB classification, which is relatively common in MLL-rearranged AML. Furthermore, splenomegaly in the mice engrafted with the MLL-AF10/K-ras^G12V^ co-transduced human HSCs was observed 8 weeks after transplantation (Figure 4D), and the average weight of their spleens was 5 times greater than in the other groups (Figure 4D). By 10 weeks after transplantation, 100% of the mice that had received MLL-AF10/K-ras^G12V^ co-transduced HSCs were dead, while all the mice in the other 3 groups survived and remained healthy 25 weeks after transplantation (Figure 4A).

We next investigated by FACS the composition of the blood cells in the BM, spleen, and liver of the MLL-AF10/K-ras^G12V^-expressing HSC-treated mice. Most of the hematopoietic cells in these tissues were GFP^+^ Venus^+^ double positive (Figure 5A). The double-positive cells were confirmed to express both the MLL-AF10 and K-ras^G12V^ genes by RT-PCR with specific primers for the exogenously transduced genes (Figure 5B). Interestingly, no cells expressing MLL-AF10 alone (GFP^+^ Venus^-^) or K-ras^G12V^ alone (GFP^−^Venus^+^) were observed in any mice in the co-transduced group, even though MLL-AF10 alone could effectively promote multilineage hematopoiesis (Figures 1 and 2). More importantly, in the group receiving K-ras^G12V^ alone-transduced HSCs, there were no Venus^+^ K-ras^G12^-expressing blood cells in any tissues examined, even in the BM (data not shown). These results indicate that the co-expression of MLL-AF10 and K-ras^G12V^ (GFP^+^Venus^+^) was necessary for the *in vivo* induction of leukemia from human HSCs.

Additional FACS data showed that the GFP^+^Venus^+^ human CD45^+^ blood cells in the all recipient mice transfused with the co-transfected HSCs had a uniform surface marker profile, CD33^+^CD11b^+^HLA-DR^+^CD14^+^CD15^+^ (Figure 5C), which was fully compatible with the FAB M5 phenotype. This finding suggested that the abnormal blood cells observed in the mice receiving co-transduced HSCs were leukemia cells. We next examined the cellular clonality of the abnormal blood cells by Southern blotting. As shown in Figure 5D, an EGFP probe that recognizes both EGFP and Venus (because the nucleotide sequences between EGFP and Venus are 98% identical) revealed one or two bands in the DNA of the abnormal leukocytes from individual mice in the co-transduced group, indicating the monoclonality of the abnormal leukocytes.

Taken together, we have successfully established a human leukemia model in which acute monoblastic leukemia cells are derived from human normal HSCs *in vivo*.

### Pathological Phenotypes of the Leukemia

Pathological examination of our human leukemia model showed that the spleens were extensively infiltrated with human hematopoietic cells, and the architecture of the red pulp and the white pulp was disrupted (Figures 6A and 6B). The tibia bones looked very pale (data not shown), and the BM was occupied by uniform blood cells, which expressed human CD45 (Figures 6A and 6B). The periportal regions of the liver were also massively infiltrated with human hematopoietic cells (Figure 6A and 6B). Hepatosplenomegaly is a common symptom in patients with acute monoblastic leukemia with rearranged MLL genes, and leukemia cell infiltration into the intrahepatic periportal regions is also a common pathological manifestation of human MLL-rearranged monoblastic leukemia. Therefore, our model may be a good representation of human MLL-rearranged monoblastic leukemia.

## Discussion

A detailed understanding of leukemogenesis requires the development of experimental murine models that can accurately mimic this process. Some studies have sought to recapitulate the harboring of MLL fusion genes in human AML using mouse HSCs [6]. However, the leukemia occurring in mice often does not faithfully recapitulate human leukemia, in part because of the biological differences between human and mouse HSCs [8], [27]. In two improved models, the MLL-ENL or MLL-AF9 gene was recently shown to be capable of initiating human leukemogenesis in NK-cell-depleted NOD/SCID mice [18] or human cytokine transgenic NOD/SCID mice [19]. However, overexpression of the MLL-AF4 fusion gene in human CD34^+^ cells is not sufficient to initiate leukemia [20]. Similar to the latter report, we found that MLL-AF10 was insufficient to induce leukemogenesis at least by 25 weeks after transplantation in the present model. In our experiments, the infectious efficiency of the MLL-AF10 vector in human HSCs was low (<2.0%). Although the efficiency of MLL-ENL and MLL-AF9 in the former report was unclear [18], differences in the infectious efficiency of the vectors used among these studies might have been responsible for the different leukemogenic effects. In another model with MLL-AF9 [19], human SCF, GM-CSF, and IL-3 were transgenically expressed in the recipient NOD/SCID mice. These cytokines may provide the MLL-AF9-transdused HSCs with additional signals for cell growth and survival, which might work as an oncogenic promoter. Comparing our model with this model, ectopic expression of K-ras^G12V^ in our model may in part compensate the cytokine (SCF, GM-CSF, and IL-3) signals in the MLL-AF9 model since stimulation with SCF and cytokines directly activates the ras signaling pathway in leukemogenesis [28]. However, it is also likely that each MLL-rearranged gene (MLL-ENL, MLL-AF4, MLL-AF9, and MLL-AF10) has a different leukemogenic efficacy, because the different partners of MLL may confer different biological functions to the produced MLL-fusion molecule [2].

Accumulating evidence points to a multistep pathogenesis for leukemia development and progression [29], [30]. In the multi-genetic step models of leukemogenesis, particularly in the two-hit model of leukemia, the initial genetic hit often leads to abnormal cell differentiation (Type II mutation), while subsequent mutations may activate specific signaling pathways that are involved in cell growth, such as the ras/MAP kinase pathway (Type I mutation) [3], [29], [31]. Indeed, MLL-rearranged AML in neonatal bloodspots was shown to be of prenatal origin, supporting the idea that MLL rearrangement is one of the earliest events (first hit) in leukemogenesis [32]. In this line, the effect of MLL-AF10 as the first hit is thought to be differentiation arrest or the promotion of a specific hematopoietic lineage, because two previous studies demonstrated that MLL-AF10 expression in mouse HSCs results in skewed myeloid hematopoiesis [21], [22]. However, in human HSCs, MLL-AF10 did not give rise to skewed hematopoiesis, but enhanced the growth (or survival) of hematopoietic cells (Figures 1B and 2). Therefore, the biological impact of MLL-AF10 as the first hit remains controversial.

The active mutation of ras genes is a known additional hit in MLL-rearranged leukemia [27], [29], [33], and was so in our model. The hematopoietic cells undergoing the first hit must survive until the additional hit occurs during leukemogenesis. Since no HSCs transdused with K-ras^G12V^ alone could be found in the BM of the humanized mice even 25 weeks after transplantation (data not shown), it is unlikely that the ras mutation was the first hit in our model. Based on the present results, MLL-AF10 as the first hit might alter the self-renewal regulation of HSCs because of its promotion effect on multi-lineage hematopoiesis (Figures 1B and 2), and the HSCs (or leukemic precursor cells) might then receive the additional hit (K-ras^G12V^), leading to leukemia. A recent report similarly demonstrated that co-transduction of BMI1 and Bcr-Abl oncogenes in HSCs induced leukemia, in which ectopic expression of BMI1 probably functioned as the additional hit, in NOD/SCID mice [34]. Although further examination will be required to test the additional hit hypothesis in leukemogenesis, our leukemia model may be a useful and unique experimental system with which to examine the multi-hit model of leukemogenesis.

Previous studies in which primary human leukemia cells were transplanted into immunodeficient mice provided important information about leukemia biology, in particular, about the biological significance of leukemic stem cells. Ishikawa et al recently demonstrated using NSG mice that the CD34^+^CD38^-^ cell population of the donor human leukemia most easily expands *in vivo* and survives by associating with the osteoblast-like stroma cells [9], [10]. These findings shed light on how the leukemic stem cells are maintained *in vivo*. However, evaluating the transplantability or tumorigenic potential of each subpopulation from primary leukemia into immunodeficient mice might be insufficient to define leukemic stem cells. Intriguingly, a recent report demonstrated using primary human melanoma cells that most of the cells (putative non-stem cells) that were not transplantable in NOD/SCID mice, which have NK cells, could easily survive and expand, like stem cells, in NSG mice, which lack NK cells [35]. This indicates the possibility that the high transplantability of the putative stem cell population as defined in the NOD/SCID mice reflected simply their resistibility to NK-cell-killing. Therefore, the observations in a certain transplantation model using immunodeficient mice may not necessarily reflect the physiological pathogenesis of human leukemic stem cells.

In this context, the leukemia that arose *in vivo* from human HSCs as demonstrated here may better reflect the physiological biology of human leukemic stem cells. Furthermore, the primary leukemia cells from different patients with leukemia showing even the same phenotypic markers should be different. Thus, the *in vivo* results from mice receiving transplants of different donor cells may be difficult to interpret, even if the leukemia cells from individual donors show the same phenotype. In contrast, the M5 leukemia established here was uniform in terms of cell morphology, symptoms (including selective tissue infiltration), and even survival prognosis. Importantly, the high reproducibility by which the same gene combination (MLL-AF10 and K-ras^G12V^) induced the same leukemia in individual mice may represent a considerable advantage of these mice for the *in vivo* modeling of human leukemia over previous models.

To examine whether the leukemia is transplantable in a second recipient, we transplanted the leukemic cells from the spleen and BM to healthy adult NOG mice at least 20 times. Unexpected, we, however, could not find any GFP^+^ cells in any second recipients even 10 months after transplantation (data not shown). Since GFP^+^Venus^+^ leukemic cells in the BM of the first hosts contained less than 0.1% CD34^+^CD38^-^ putative AML stem cells (data not shown), leukemic stem cells might have lost their self-renewal ability and been run out in the first host. To understand the unexpected phenomenon, Further investigation will be required.

Over the last decade, the outcome in pediatric AML has improved significantly, with up to 60% of children suffering from MLL-rearranged AML currently surviving [3]. However, improving the outcome of pediatric AML using current treatment protocols is hampered by treatment-related deaths and long-term side effects. In addition, most patients with MLL-rearranged ALL still indicate a poor prognosis. Therefore, to improve the outcome in pediatric MLL-rearranged leukemia, the development of leukemia-specific targeting drugs is an important strategy [36]. We hope that this newly established model using humanized mice will contribute to future studies aimed at revealing the molecular mechanisms for MLL-rearranged gene related leukemogenesis and developing new therapies against MLL-related malignancies.
